# Supplementary material for: The Systems Biology Research Tool: evolvable open-source software
Source: BMC Syst Biol. 2008 Jun 29;2:55. doi: 10.1186/1752-0509-2-55 (PMC2446383; doi:10.1186/1752-0509-2-55)
Supplement: Additional file 1 — SBRT Archive. An archive of the current version of the Systems Biology Research Tool. [file 1752-0509-2-55-S1.zip › sbrt-1.4.0/doc/developers_guide/api/sbrt/kernel/util/ProgressMonitor.html]

ProgressMonitor


|  |  |  |
| --- | --- | --- |
| **Overview** \| **Package** \| **Class** \| **Use** \| **Tree** \| **Deprecated** \| **Index** \| **Help** | |  |
| **PREV CLASS**   **NEXT CLASS** | **FRAMES**    **NO FRAMES**     **All Classes** |  |
| SUMMARY: NESTED \| FIELD \| CONSTR \| METHOD | DETAIL: FIELD \| CONSTR \| METHOD |  |


---


## sbrt.kernel.util Interface ProgressMonitor

**All Known Subinterfaces:**: MutableProgressMonitor

**All Known Implementing Classes:**: AbstractProgressMonitor, DiscreteProgressMonitor, PercentProgressMonitor

---

``` public interface ProgressMonitor ```

This interface is used to represent progress monitors.

**Author:**
:   This interface was written and documented by
    Jeremiah Wright while in the Wagner lab.

---

| **Method Summary** | |
| --- | --- |
| `long` | `getDuration()`             Returns the current duration of the process being monitored. |
| `java.lang.Number` | `getProgress()`             Returns a number indicating the progress of the process being monitored. |
| `boolean` | `started()`             Indicates if this progress monitor has already been started. |
| `boolean` | `stopped()`             Indicates if this progress monitor has already been stopped. |

**Method Detail**

### getDuration

```
long getDuration()
```

:   Returns the current duration of the process being monitored.

    :   **Returns:**: the current duration of the process in milliseconds.

---


### getProgress

```
java.lang.Number getProgress()
```

:   Returns a number indicating the progress of the process being monitored.

    :   **Returns:**: a number indicating the progress of the process being monitored.

---


### started

```
boolean started()
```

:   Indicates if this progress monitor has already been started.

    :   **Returns:**: `true` if this progress monitor has already been started; `false` otherwise.

---


### stopped

```
boolean stopped()
```

:   Indicates if this progress monitor has already been stopped.

    :   **Returns:**: `true` if this progress monitor has already been stopped; `false` otherwise.


---


|  |  |  |
| --- | --- | --- |
| **Overview** \| **Package** \| **Class** \| **Use** \| **Tree** \| **Deprecated** \| **Index** \| **Help** | |  |
| **PREV CLASS**   **NEXT CLASS** | **FRAMES**    **NO FRAMES**     **All Classes** |  |
| SUMMARY: NESTED \| FIELD \| CONSTR \| METHOD | DETAIL: FIELD \| CONSTR \| METHOD |  |


---
